# Milk‐Clotting Protease From *Bacillus stercoris* NCCP‐3139: A Potential Microbial Rennet for Cheese Production

**DOI:** 10.1002/fsn3.71864

**Published:** 2026-05-13

**Authors:** Muhammad Sibtain, Ashraf Ahmed Qurtam, Sadaf Javaria, Ghulam Murtaza, Mohammed Al‐zharani, Noor‐ul Ain, Sadia Chaman, Fahd A. Nasr, Ali Zaman

**Affiliations:** ^1^ Institute of Food Science and Nutrition Gomal University Dera Ismail Khan Pakistan; ^2^ Biology Department, College of Science Imam Mohammad Ibn Saud Islamic University (IMSIU) Riyadh Saudi Arabia; ^3^ Faculty of Veterinary and Animal Sciences (FVAS) Gomal University Dera Ismail Khan Pakistan; ^4^ Institute of Pharmaceutical Sciences UVAS Lahore Pakistan; ^5^ Institute of Microbiology (FVAS) Gomal University Dera Ismail Khan Pakistan

**Keywords:** *Bacillus* species, casein hydrolysis, milk‐clotting activity, purification, thermostable protease

## Abstract

Milk‐clotting enzymes (MCEs) are essential biocatalysts in cheese manufacture. However, limited availability and ethical concerns associated with calf rennet have intensified the search for microbial alternatives. This study reports the purification and characterization of a milk‐clotting protease from *Bacillus stercoris* NCCP‐3139, a soil isolate from Dera Ismail Khan, Pakistan. The strain was identified as *B. stercoris* by standard morphological and biochemical assays and 16S rRNA gene sequencing (98.75% similarity to *B. stercoris* JCM 30051). The enzyme was purified by ammonium sulfate precipitation, dialysis, DEAE–cellulose ion‐exchange chromatography and Sephacryl S‐200 gel filtration chromatography. Milk‐clotting activity was assessed in goat, sheep, cow, buffalo and camel milk. The isolate exhibited pronounced casein hydrolysis and significant milk‐clotting activity (*p* < 0.05), consistent with the production of a rennin‐like protease. Sheep and buffalo milk showed superior clotting performance compared with other species (*p* < 0.05). Purification increased specific activity from 1.6 to 5.0 U/mg and the milk‐clotting‐to‐proteolytic activity (PA) ratio from 50 to 550, indicating significant catalytic selectivity. The enzyme displayed optimal activity at 55°C and pH 9.0, and was activated by Mn^2+^ and Sr^2+^ but strongly inhibited by EDTA, Hg^2+^ and Zn^2+^. SDS–PAGE revealed a single band, consistent with a monomeric enzyme. Thus isolated *B. stercoris* NCCP‐3139 produces protease with significant specificity and selectivity indicating its potential as a microbial alternative to animal rennet in cheese production.

## Graphical abstract

A milk‐clotting protease from *Bacillus stercoris* NCCP‐3139 was isolated from soil and purified via ammonium sulfate precipitation and sequential chromatographic techniques, yielding a homogeneous enzyme confirmed by SDS–PAGE. The protease exhibited high catalytic selectivity, with a marked increase in milk‐clotting‐to‐proteolytic activity ratio (50–550) and broad activity across milk types, showing superior performance in sheep and buffalo milk. Its optimal activity at 55°C and alkaline pH, along with metal ion responsiveness, underscores its potential as a robust microbial rennet alternative for cheese production.

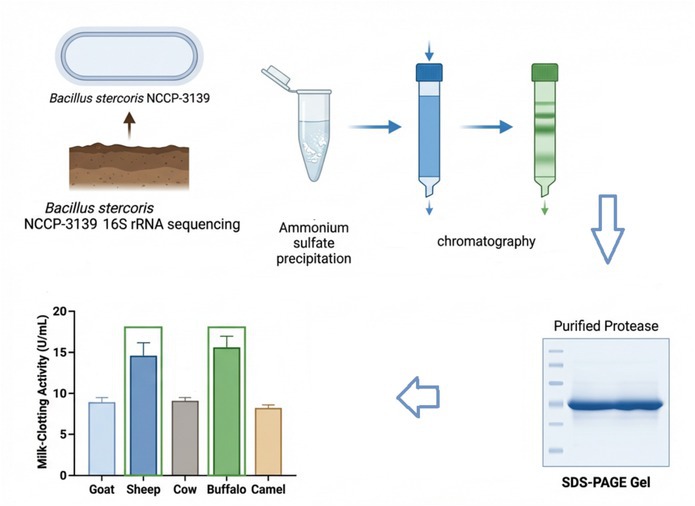

## 1 Introduction

Proteolytic enzymes are important biocatalysts that are widely used in industrial processes across the dairy, food, and pharmaceutical industries. Among these, MCEs play a crucial role in cheese production, as they hydrolyze κ‐casein in milk, initiating coagulation and curd formation (Bilal and Iqbal 2020). Traditionally, animal‐derived rennet has been used for coagulating milk; however, rising global cheese demand, ethical concerns about animal slaughter, and the availability of microbial and plant‐based rennets have prompted research into microbial and plant‐based alternatives. Microbial proteins, particularly those from *Bacillus* species, have been proposed as promising alternatives because they are highly productive, stable, and amenable to genetic manipulation (Zhang, Tao, et al. 2025; Zhang, Yang, et al. 2025).

*B. stercoris* is a soil‐dwelling bacterium, and is characterized by the secretion of thermostable and alkaline proteases with potential applications in food bioprocessing. Protease enzymes are used industrially due to their significant catalytic properties across a wide range of pH and temperature (Banerjee and Ray 2017). Microbial‐MCEs with a high milk‐clotting‐to‐proteolytic activity ratio (MCA/PA) are necessary to reduce nonspecific proteolysis, which may adversely affect cheese texture and yield. Thus, screening, purification, and characterization of novel *Bacillus*‐derived enzymes are important for identifying effective and cost‐effective biocatalysts and dairy products (Ali et al. 2025).

Given the increasing demand for cheese and the limitations associated with animal‐derived rennet, microbial milk‐clotting enzymes have gained attention as scalable and potentially sustainable alternatives (Priyashantha 2025). Among these, *Bacillus*‐derived enzymes are of particular interest because they can be produced by fermentation and may provide favorable stability and catalytic properties for dairy processing (Raman et al. 2025; Kumar et al. 2025).

The main aim of this research is to isolate, purify, and characterize a milk‐clotting protease from *Bacillus stercoris* NCCP‐3139, focusing on its milk‐clotting‐to‐proteolytic activity ratio (MCA/PA), optimal physicochemical conditions, and commercial potential. The study underscores its potential as an eco‐friendly and cost‐effective alternative to animal‐based rennet.

## 2 Materials and Methods

### 2.1 Study Area

This study was carried out at the Institute of Microbiology and the Institute of Food Science and Nutrition, Gomal University, Dera Ismail Khan, Pakistan.

### 2.2 Isolation of Bacterial Strain

Soil samples were aseptically collected from the vicinity of the Institute of Microbiology, Gomal University (31.8188° N, 70.8971° E). A total of 10 soil samples were collected, each from a depth of 5–10 cm and afterwards transported in sterile polythene bags at 4°C. One gram of soil was serially diluted in sterile 0.9% saline, and dilutions were spread on R2A agar medium (0.6 g peptone, 0.6 g yeast extract, 0.6 g glucose, 0.6 g casamino acids, 0.6 g soluble starch, 0.3 g sodium pyruvate, 0.3 g K_2_HPO_4_·7H_2_O, 0.005 g MgSO_4_·7H_2_O, 15 g agar, pH 7.0). Plates were incubated at 37°C for 48 h. Distinct colonies were purified by streaking and maintained on nutrient agar at 4°C. For long‐term storage, cultures were preserved in 20% glycerol at −70°C (Nair et al. 2021).

### 2.3 Morphological and Biochemical Characterization

#### 2.3.1 Colony Morphology

Colony size, shape, margin, elevation, surface texture, and pigmentation were recorded from nutrient agar plates (Taredahalli 2013).

#### 2.3.2 Microscopy and Staining

Gram staining, endospore staining (malachite green/safranin), and motility testing in semi‐solid agar were performed following standard protocols (Ryu et al. 2024).

#### 2.3.3 Biochemical Tests

Biochemical tests included methyl red (MR), Voges–Proskauer (VP), urease, catalase, citrate utilization, starch hydrolysis, casein hydrolysis, and indole test (Al‐Joda and Jasim 2021).

### 2.4 Molecular Characterization

#### 2.4.1 DNA Extraction

Genomic DNA was extracted using the SDS–phenol–chloroform–isoamyl alcohol method. DNA was dissolved in TE buffer and stored at −20°C (Rath and Das 2023).

#### 2.4.2 PCR Amplification of 16S rRNA Gene

PCR amplification of the 16S rRNA gene was performed using universal primers 27F and 1492R. Amplified products (~1116 bp) were visualized on 1.8% agarose gel stained with ethidium bromide (Satilmis et al. 2019).

#### 2.4.3 Sequencing and Phylogenetic Analysis

PCR products were sequenced commercially (NCCP, Islamabad, Accession Id PV363620). Sequences were compared with NCBI GenBank and EzBioCloud databases using BLAST. Multiple sequence alignment was performed with CLUSTAL_X, and a neighbor‐joining phylogenetic tree was generated in MEGA 7.0 with 1000 bootstrap replications (Chouaia and Dittmer 2024).

### 2.5 Milk Sampling and Composition Analysis

Fresh cow (*n* = 15), buffalo (*n* = 15), sheep (*n* = 15), goat (*n* = 20), and camel (*n* = 10) milk samples were aseptically collected from local farms in Bhakkar and Layyah districts. Milk samples were collected from clinically healthy animals at mid‐lactation (or near to mid) to minimize physiological variation associated with early‐ and late‐lactation changes in milk composition and coagulation behavior (Visentin et al. 2017). Immediately after collection, milk samples from each species were pooled and kept at 4°C prior to proximate composition analysis. Proximate composition (protein, fat, lactose, solids‐not‐fat, total solids, and ash) was determined. The Fat, protein, lactose, total solids, and solids‐not‐fat were measured using Ultrasound Milk Analyzer (Milkotronic Ltd. Lactoscan SA50), whereas ash content was determined by protein by the Kjeldahl method according to a previously described method by Stocco et al. (2025).

### 2.6 Enzyme Production and Extraction

Enzyme production was carried out in 250 mL Erlenmeyer flasks containing 100 mL of sterile skim milk medium (cow, buffalo, sheep, goat, or camel milk) by removal of the fat fraction prior to sterilization. Raw milk from each species was first defatted to prepare skim milk. Briefly, milk samples were centrifuged at 5000 × g for 15 min at 4°C, the cream layer was removed aseptically, and the resulting skim fraction was used as the casein‐containing substrate in the fermentation medium prior to sterilization and inoculation. The skim milk was used as the primary casein‐containing substrate, whereas corn steep liquor and the added salts (K_2_HPO_4_, (NH_4_)_2_HPO_4_, and MgCl_2_) were included to support microbial growth, maintain nutrient balance, and promote protease production. The initial inoculum density was approximately 10^6^ CFU/mL, as determined by optical density (OD) measurements at 600 nm or by plate counting (colony‐forming units per mL). The cultures were incubated at 37°C with continuous shaking at 150 rpm for 24–120 h. The pH of the medium was not actively controlled during fermentation, but it was monitored periodically. The pH was observed to fluctuate between 7.0 and 8.0 during the fermentation process, depending on the species of milk used (Wang et al. 2025).

### 2.7 Protease Activity Assay

PA was determined using casein as substrate. The reaction was terminated with 5% trichloroacetic acid, centrifuged, and the absorbance was measured at 650 nm. One unit of PA was defined as the amount of enzyme that releases 1 μg of tyrosine per min. Protein concentration was estimated by the Lowry assay (Zhang, Tao, et al. 2025; Zhang, Yang, et al. 2025). It was calculated as follows:

$$\mathrm{PA}\;\left(U/\mathrm{mL}\right)=\frac{\text{Amount of tyrosine released}\;\left(\mathrm{\mu g}\right)}{\text{Time}\;\left(\min\right)\times \text{Volume of sample}\;\left(\mathrm{mL}\right)}$$

### 2.8 Milk‐Clotting Activity (MCA)

The cultures were incubated at 37°C with shaking at 150 rpm for 24, 48, 72, 96, and 120 h to allow enzyme production. At each time point, culture supernatants were collected aseptically and used as crude enzyme preparations for the milk‐clotting assay. At each incubation time point, culture supernatants were collected aseptically and used as crude enzyme preparations. Milk‐clotting activity was then determined separately using reconstituted skim milk containing 0.1 M CaCl_2_ at 37°C, and clotting was monitored for up to 40 min. One unit of MCA was defined as the amount of enzyme required to clot 10 mL of milk at 37°C within 40 min (Jasim et al. 2025). The MCA was calculated as follows:

$$\mathrm{MCA}\;\left(U/\mathrm{mL}\right)=\frac{\text{Volume of milk clot}\;\left(\mathrm{mL}\right)}{\text{Time}\;\left(\min\right)}$$

### 2.9 MCA/PA Ratio

The ratio of milk‐clotting activity (MCA) to proteolytic activity (PA) is an important parameter for evaluating enzyme selectivity. The MCA/PA ratio is calculated by dividing the MCA by the PA, as follows:

$$\mathrm{MCA}/\mathrm{PA}\;\text{ratio}=\frac{\;\mathrm{MCA}\;\left(U/\mathrm{mL}\right)}{\mathrm{PA}\;\left(U/\mathrm{mL}\right)}$$

### 2.10 Enzyme Purification

The crude enzyme solution was precipitated at 4°C with ammonium sulfate at 30%–70% saturation in two steps (30%–50% and 50%–70%), followed by centrifugation at 10,000 rpm for 10 min and resuspension in phosphate buffer (pH 7.5) containing 50 mM NaCl. The solution was dialyzed to remove ammonium sulfate. For further purification, DEAE‐cellulose ion exchange chromatography was performed using 20 mM Tris–HCl (pH 7.5) and 50 mM NaCl (Buffer A), with elution via a linear NaCl gradient (50 mM to 500 mM). The enzyme was then subjected to Sephacryl S‐200 gel filtration chromatography using the same buffer. The flow rate for DEAE‐cellulose was 1 mL/min, with fractions collected in 5 mL volumes, and for Sephacryl S‐200, the flow rate was 0.5 mL/min, with fractions collected in 2 mL volumes. Protein content was monitored at 280 nm, and fractions with the highest specific activity were pooled (Bytyçi et al. 2025).

### 2.11 Effect of Physicochemical Parameters

The effect of pH (5–11), temperature (30°C–80°C), and metal ions/inhibitors (Mn^2+^, Sr^2+^, Ca^2+^, Zn^2+^, Hg^2+^, K^+^, SDS, EDTA) on enzyme activity was studied as described by (Im et al. 2025).

### 2.12 SDS–PAGE and Zymography

Enzyme purity and molecular weight were analyzed by SDS–PAGE. Casein zymography was used to confirm proteolytic activity (Acharjee et al. 2025).

### 2.13 Statistical Analysis

All experiments were performed in triplicate, and results are presented as mean ± standard deviation (SD). Data were analyzed using one‐way ANOVA followed by Tukey's test in GraphPad Prism 9.0. Statistical significance was considered at *p* < 0.05. The experimental units for each comparison were independent biological replicates, with each fermentation or enzyme preparation conducted in a separate flask and treated as an individual sample. The assumptions of normality and homoscedasticity were assessed prior to performing ANOVA. Normality was assessed using the Shapiro–Wilk test, and homoscedasticity was evaluated using Levene's test.

## 3 Results

### 3.1 Morphological Characterization

The isolate *Bacillus stercoris* NCCP‐3139 exhibited typical *Bacillus* characteristics, including the formation of irregular, opaque colonies with rough margins and a Gram‐positive, endospore‐forming, rod‐shaped morphology (Figure S1). Biochemical tests, as summarized in Table 1, were positive for methyl red, urease, catalase, and starch hydrolysis, consistent with *Bacillus*. These findings confirm the strain's identity and support its potential for protease production, which is relevant to industrial applications. Gram‐positive cells that were observed under a microscope were in the form of rods (Figure S1). Endospore staining supported the interpretation that green endospores were present within pink vegetative cells, indicating that the strain forms spores. Moreover, the strain was motile as shown by the diffuse growth of the stab line in semi‐solid agar (Figure S1). Based on data outcomes, it was evident that the isolated strain was representative of the genus *Bacillus*.

**TABLE 1:** Biochemical characterization of *B. stercoris* NCCP‐3139.

S. No	Biochemical characterization of *B. stercoris* NCCP‐3139		
	Biochemical test	Observation	Result
1	MR test	Red color	+
2	VP test	No cherry red color	−
3	Urease test	Deep pink color	+
4	Catalase test	Bubble formation	+
5	Citrate utilization	Green to blue color change	+
6	Starch hydrolysis	Clear hydrolytic zone	+
7	Indole test	No red color	−

### 3.2 Biochemical Characterization

The *B. stercoris* NCCP‐3139 was further identified in biochemical analysis. The isolate produced a positive methyl red (MR) reaction, suggesting stable acid production from glucose fermentation, whereas the Voges–Proskauer (VP) test was negative, indicating the absence of acetoin formation. The strain exhibited strong urease and catalase activities, confirmed by deep pink coloration and vigorous effervescence, respectively. The presence of positive citrate was indicated by a color change to blue, whereas starch hydrolysis produced a clear halo around the colonies, signifying amylase activity. Conversely, the indole test was negative, and no red color was observed upon addition of Kovac's reagent (Table 1). The outcomes were consistent with earlier investigations. For example, the successful isolation of thermostable proteases from *Bacillus* species supports the feasibility of this approach for obtaining high‐activity enzymes suitable for industrial applications (Ibrahim and Ma 2017). Additionally, various investigators have shown that the use of soil samples for isolating *Bacillus* strains is a common and effective strategy for obtaining proteases with desirable properties for dairy applications (Khatoon et al. 2023; Patil and Jadhav 2017).

### 3.3 Casein Hydrolysis and Milk‐Clotting Activity

*B. stercoris* NCCP‐3139 was found to present “strong hydrolysis” of casein, and a clearance zone was produced with a large size on skim milk agar (Figure S1). The “strong” hydrolysis was identified based on the speed of coagulation as described earlier (Chen et al. 2021). This confirmed that extracellular proteases capable of degrading casein were secreted (Baggiolini et al. 2009). The milk‐clotting assay showed high activity (*p* < 0.05) compared with non‐proteinolytic strains, indicating the enzyme's ability to cleave κ‐casein, a fundamental step in micelle destabilization and milk coagulation (Ben Amira et al. 2017). These results indicate that *B. stercoris* NCCP‐3139 expresses a rennin‐like protein with strong potential for industrial cheese production. The ratio of milk‐clotting activity (MCA) to proteolytic activity (PA) is critical in determining the efficiency and suitability of microbial coagulants for cheese production (Moschopoulou 2017).

### 3.4 Molecular Identification

The isolate was further confirmed by molecular tests, which involved amplification of the 16S rRNA gene, yielding a single band of approximately 1116 bp on agarose gel electrophoresis (Figure S2). The phylogenetic tree based on 16S rRNA gene sequences was constructed using the Molecular Evolutionary Genetics Analysis (MEGA software) employing the Neighbor‐Joining method with bootstrap analysis. The taxonomic identity of the isolate was established by sequence analysis (Table 2), showing it to be similar to *B. stercoris* JCM 30051 (98.75%). The NCCP‐3139 clustering with a separate one that was closely related to strain *B. stercoris* JCM 30051 with a bootstrap value of 71% (Figure 3). This phylogenetic relationship supports the taxonomic identification of the isolate and confirms its close evolutionary affinity with *B. stercoris* . This approach helps identify the strain's taxonomic position and assess its genetic diversity relative to other strains within the genus *Bacillus* (Sacchi et al. 2002).

**TABLE 2:** Molecular identification of *B. stercoris* NCCP‐3139.

Strain ID	16S rRNA size (bp)	Closely related taxa	Accession no.	Similarity %
NCCP‐3139	1116	*B. stercoris* JCM 30051	PV363620	98.75%

### 3.5 Comparative Evaluation of Milk Composition

**FIGURE 1:**
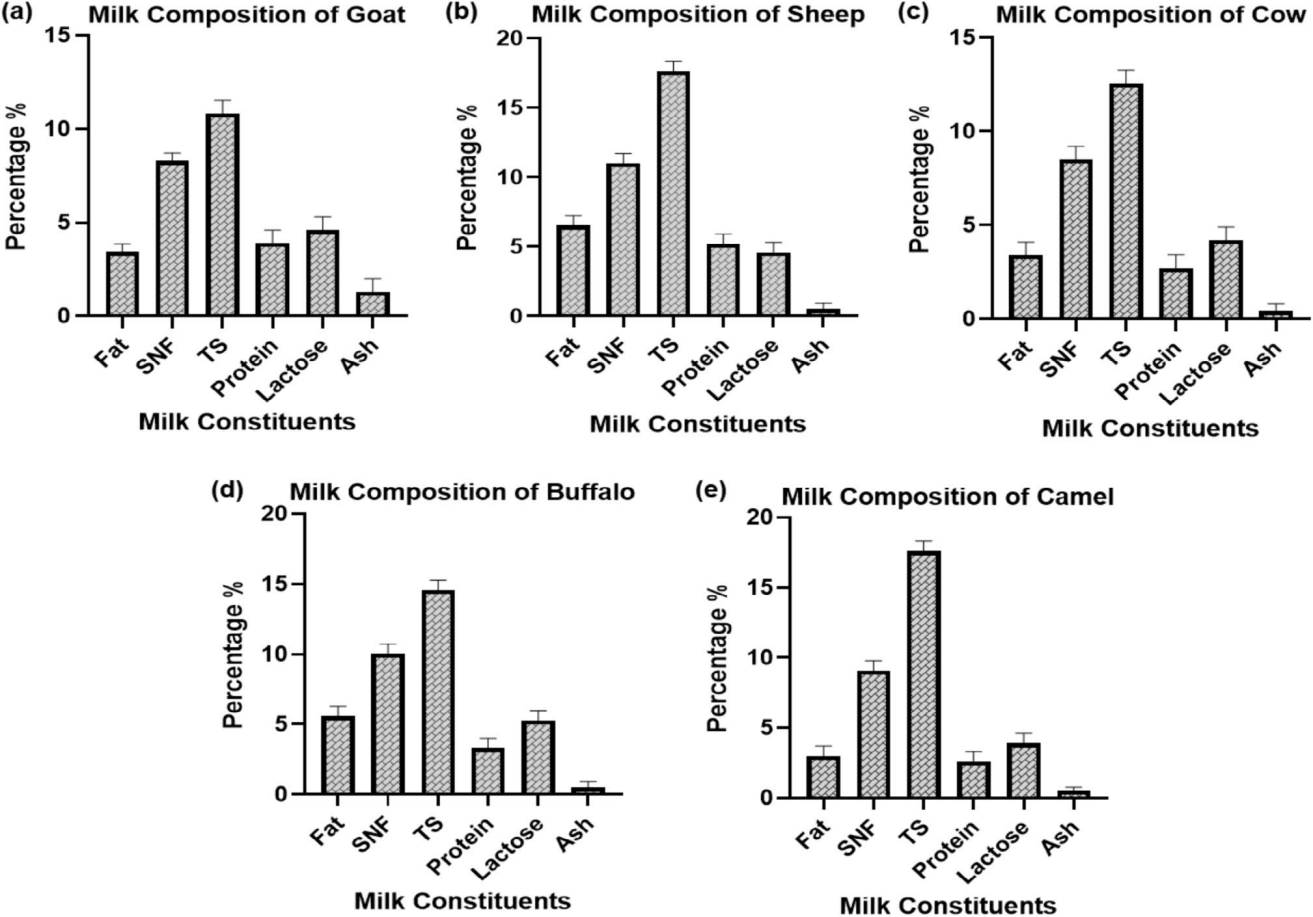
Assembly of milk composition obtained from various sources, including Goat (a) Sheep (b) Cow (c) Buffalo (d) and Camel (e).

The results of the comparative milk content of goat, sheep, cow, buffalo, and camel reporting significant differences (*p* < 0.05) in the key milk components, such as fat, SNF, TS, protein, lactose, and ash (Figure 1). Goat milk had an average content of fat and protein, with relatively lower SNF and TS, implying lower nutrient density. The highest concentrations of fat, protein, and total solids are in sheep milk (*p* < 0.05), making it more suitable for dairy processing. The intermediate levels of fat and protein in cow's milk were compensated for by high lactose levels (*p* < 0.05), which confirmed its digestibility and industrial applicability. The fat, SNF, and TS levels were significantly higher in buffalo milk (*p* < 0.05), indicating suitability for high‐fat dairy products such as butter and ghee. In contrast, camel milk exhibited lower fat and protein concentrations (*p* < 0.05) but higher lactose content, which enhances its digestibility and therapeutic benefits, particularly in arid regions. These compositional differences highlight the species‐specific nutritional and functional characteristics of the milk (*p* < 0.05).

### 3.6 Proteolytic Activity

**FIGURE 2:**
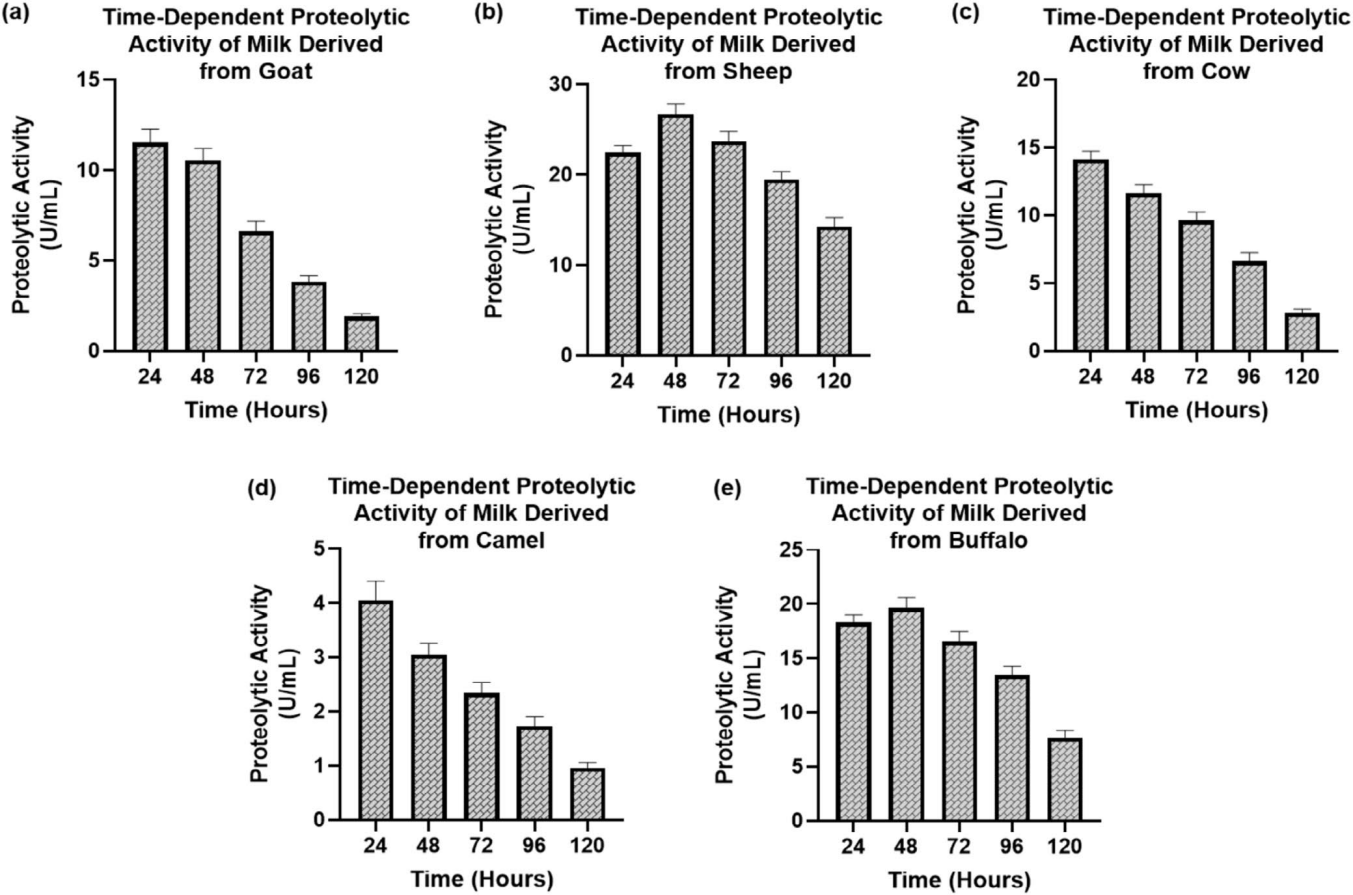
Assessment of time dependent proteolytic activity of milk obtained from various sources: Goat (a), Sheep (b), Cow (c), Camel (d), and Buffalo (e).

Proteolytic activity varied significantly among milk types and fermentation times (*p* < 0.05) (Figure 2). The enzymatic activities were the highest in sheep and buffalo milk, with 27 and 22 U/mL recorded at 48 h at the highest point, and 72 h recorded in the gradual decline, respectively. Moderate proteolytic activity was observed in goat and cow milk, with the lowest level of the same recorded in camel milk (1–3.5 U/mL) during incubations. The decreasing behavior with time (*p* < 0.05) indicates inactivation of enzymes or depletion of the substrates. These findings show that proteolytic performance is determined by the milk composition, stability of enzymes, and species‐specific protein content.

### 3.7 Milk‐Clotting Activity (MCA)

**FIGURE 3:**
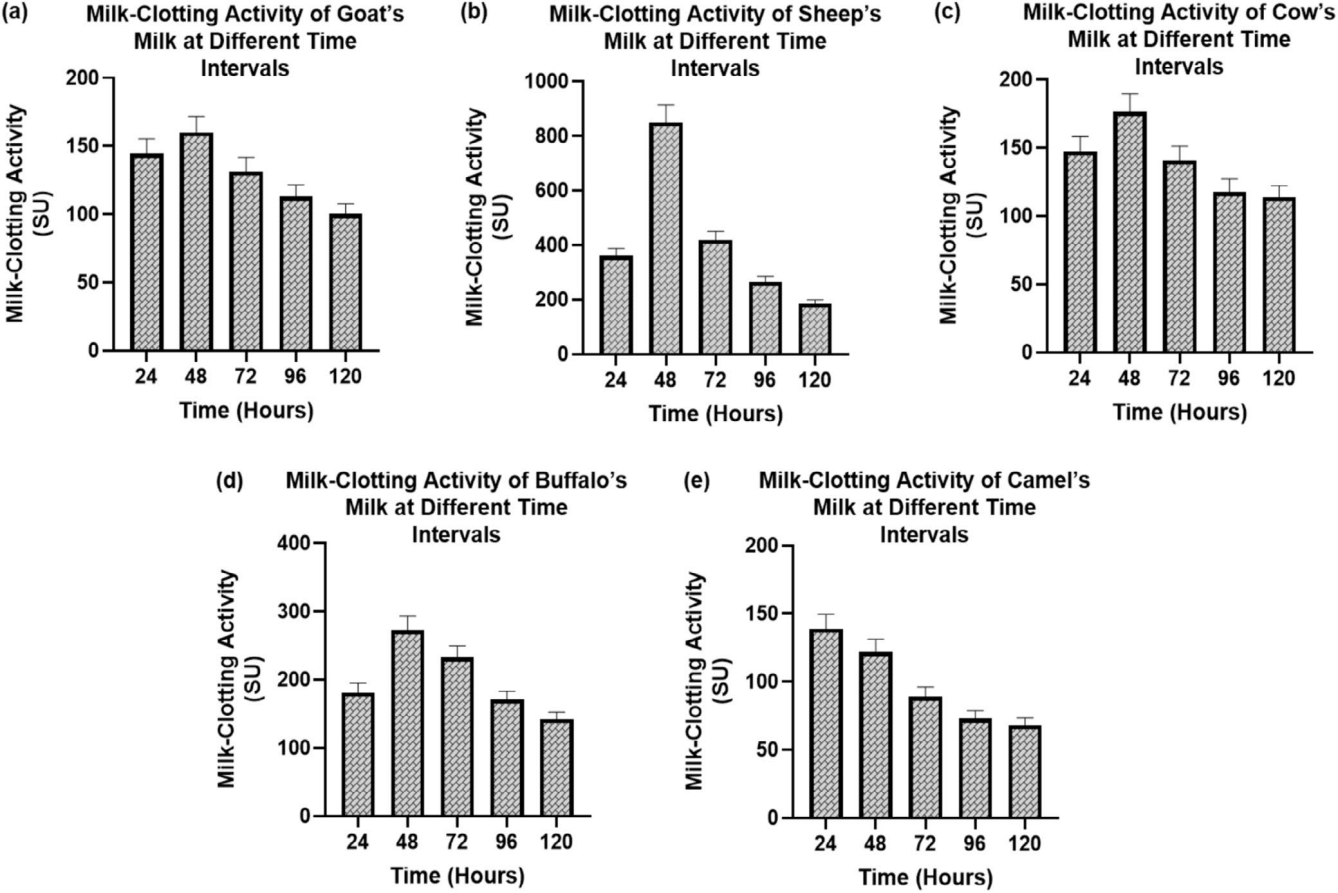
MCA of Milk‐clotting activity of crude enzyme preparations harvested after 24, 48, 72, 96, and 120 h of fermentation in media prepared from goat, sheep, cow, buffalo, and camel milk. Values are expressed as mean ± SD of three independent experiments.

There was a significant difference of MCA between the five milk types and incubation times (Figure 3). The highest content of MCA was found in sheep milk at 48 h, which was the highest (≈950 SU), then buffalo milk (≈300 SU) and cow milk (≈160 SU). Goat milk was in the middle (~160 SU at 48 h) whereas camel milk had the lowest coagulation potential (~60 SU at 120 h). The activity reduction after 48 h (*p* < 0.05) in all the species implies excessive hydrolysis of casein or enzyme destabilization. High casein and calcium content makes the superior MCA in sheep and buffalo milks put them at the forefront of industry in terms of producing cheese. MCA varied significantly among milk types and fermentation time points. The reported values correspond to enzyme preparations harvested after different fermentation times (24–120 h), whereas the clotting assay itself was performed separately under standardized assay conditions. The values shown represent the MCA of enzyme preparations harvested after 24–120 h of bacterial cultivation, rather than the duration of the clotting assay itself.

### 3.8 Enzyme Purification and Characterization

**FIGURE 4:**
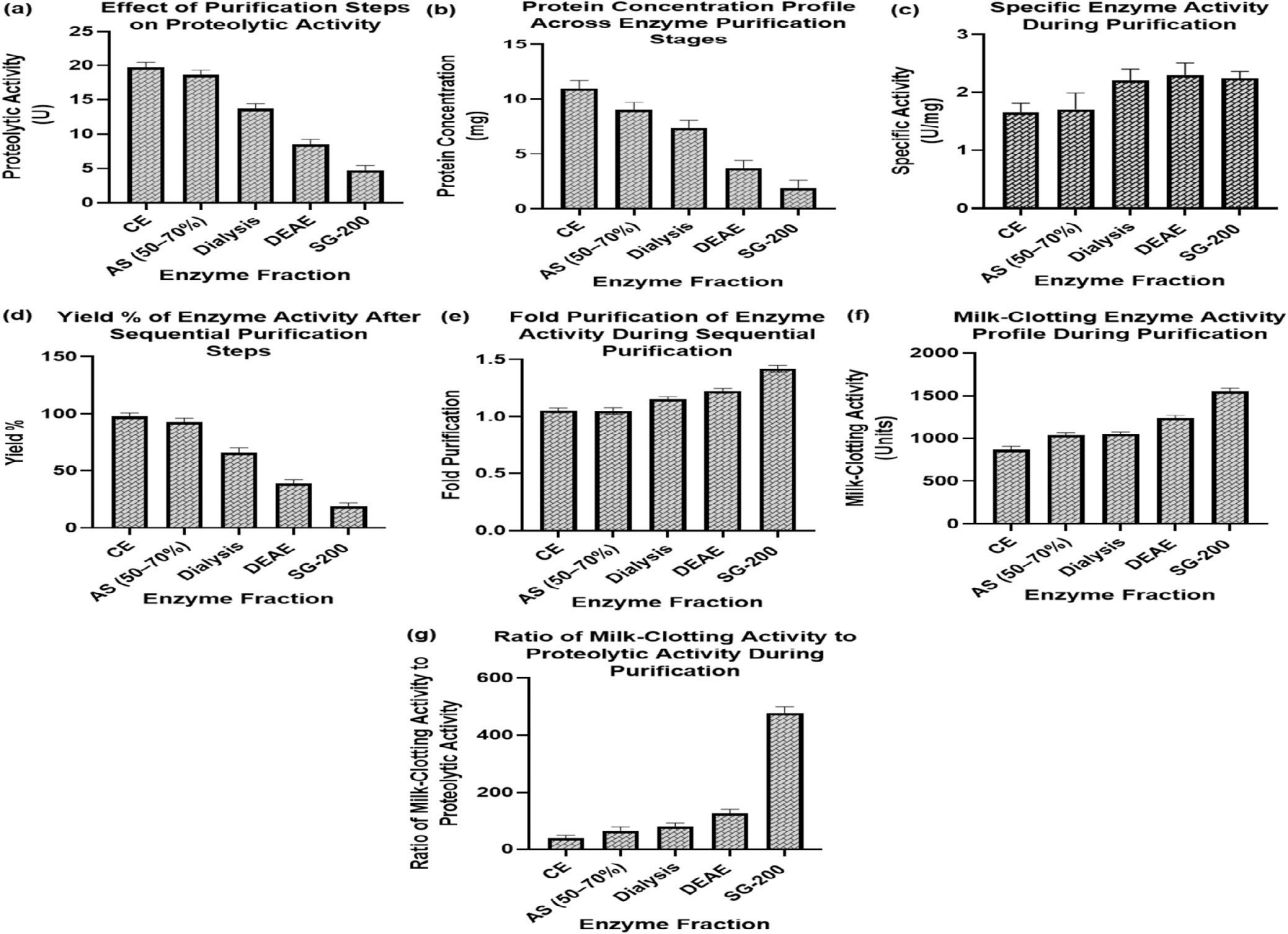
The effects of various parameters related to enzyme purification, including (a) PA, (b) total protein, (c) specific activity, (d) yield %, (e) fold purification, (f) MCA Profile, and (g) MCA/PA ratio.

The enzyme was sequentially purified by ammonium sulfate precipitation (30%–70%), dialysis, DEAE‐cellulose ion exchange, and Sephacryl S‐200 gel filtration successively (Figures S2 and S3). The proteolytic activity (PA) decreased by 22 to 10 U/mL (*p* < 0.05) and the concentration of the protein reduced significantly (*p* < 0.05) by 13 to 2 mg/mL, which indicated that non‐specific proteins were eliminated (Ullah et al. 2022; Naveed et al. 2023). Particular activity increased by significant (*p* < 0.05) values between 1.6 and 5.0 U/mg, which proved to be purified since there exist no enlarged band development (Suryani et al. 2023). The yield decreased to 25% from 100% (*p* < 0.05), a pattern characteristic of multistep purification. The fold purification increased by 0.5 and the MCA increased by 800 to 1700 SU (*p* < 0.05). The ratio of MCA‐to‐PA rose drastically between 50 and 550, showing a significant difference between clotting and hydrolyzing caseins (Figure 4). As shown in Figure 4, enzyme purification progressively reduced total protein content while increasing specific activity and the MCA/PA ratio. These results indicate that the Sephacryl S‐200‐purified enzyme is the most active and the most specific for industrial use.

### 3.9 Characterization of Purified Enzyme

#### 3.9.1 Effect of Temperature on *PA*

**FIGURE 5:**
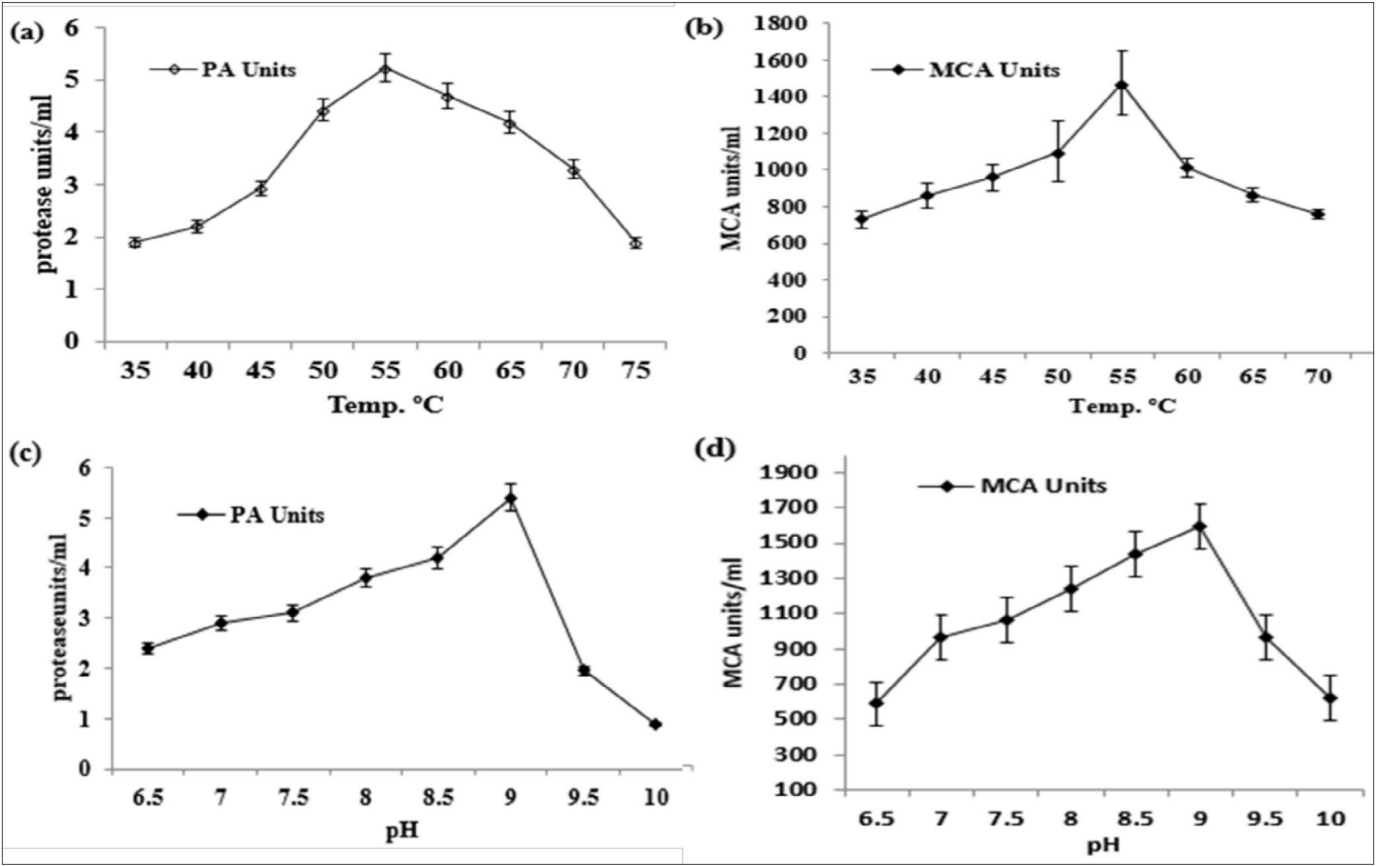
Effect of temperature and pH on the activity of the purified enzyme. (a) PA at different temperatures, (b) MC activity at different temperatures, (c) PA at different pH values, and (d) MC activity at different pH values. Values are expressed as mean ± SD. The tested ranges included 30°C–80°C for temperature and pH 5.0–11.0 for pH.

The optimal temperature for proteolytic and milk‐clotting activities was 55°C, and the optimal pH was 9.0 (Figure 5a). To confirm the statistical significance of these optima, we compared enzyme activity at neighboring temperatures and pH values (e.g., 50°C and 60°C, pH 8.5 and 9.5) using one‐way ANOVA followed by Tukey's test. The results indicated that enzyme activity at 55°C and pH 9.0 was significantly higher (*p* < 0.05) than that under adjacent conditions. Confidence intervals (or SD bars) will be provided in the figures (Figure 5) to illustrate the variability and statistical significance of these findings. At 80°C, PA further declined, confirming marked thermal inactivation beyond the optimum temperature. However, during freezing, a drastic decrease in activity was observed above 55°C, with activity decreasing to 4.7, 4.2, and 3.3 U/mL at 60°C, 65°C, and 70°C, respectively, and to 1.89 U/mL at 75°C. The decrease above the optimal temperature can be explained by denaturation and conformational alterations in the enzyme's structure. Therefore, 55°C was the highest temperature at which PA was maximized. At 80°C, only residual PA was observed, indicating marked thermal inactivation of the enzyme beyond the optimum temperature.

#### 3.9.2 Effect of Temperature on *MCA*

As shown in Figure 5b, the MCA of the purified enzyme increased gradually (*p* < 0.05) to 1475.6 SU at 55°C. At temperatures beyond this, MCA declined significantly (*p* < 0.05) to 1011.29 SU at 60°C, 865.41 SU at 65°C, and 756.4 SU at 70°C. The temperature effects indicate that 55°C is the best temperature for added coagulation of milk, where heat‐induced denaturation may have caused the instability of the catalytic site of the enzyme. Similarly, at 80°C, MCA decreased further, indicating substantial loss of catalytic efficiency at elevated temperature. The results suggest that the enzyme can be used under moderate thermal conditions, which are appropriate for the milk‐processing industry.

#### 3.9.3 Effect of pH on *PA*

The effect of pH on PA is shown in Figure 5c. It was observed that enzyme activity increased progressively (*p* < 0.05) with increasing pH, ranging from 2.4 U/mL at pH 6.5 to a maximum of 5.4 U/mL at pH 9.0. It was found that activity decreased drastically after this point where 1.95 U/mL at pH 9.5 and 0.89 U/mL at pH 10. At pH 11, PA further decreased indicating severe loss of enzyme function under strongly alkaline conditions. These findings reveal that the enzyme works best in a slightly alkaline environment (pH 9), as is characteristic of alkaline proteins. This loss of activity at higher pH is presumably due to structural destabilization or ionization of the structurally important amino acid residues required to catalyze. At pH 11, only residual PA was detected, indicating severe loss of enzyme function under strongly alkaline conditions.

#### 3.9.4 Effect of pH on *MCA*

The Figure 5d shows the influence of pH on MCA The MCA of the enzyme differed significantly (*p* < 0.05) with a maximum of 1593.16 SU at pH 9.0 being the optimum catalytic efficiency of the enzyme. At the same time, past this optimum, MCA dropped rapidly to 967.78 SU at pH 9.5 and 623.32 SU at pH 10. Likewise, at pH 11, MCA declined further confirming that catalytic performance was strongly compromised beyond the optimum pH. The fact that this enzyme prefers a high pH makes it highly beneficial in industrial cheese‐making processes that require the enzyme to remain stable at high pH. Likewise, at pH 11, MCA declined to a residual level, confirming that catalytic performance was strongly compromised beyond the optimum pH.

### 3.10 Effect of Metal Ions and Enzyme Inhibitors

**FIGURE 6:**
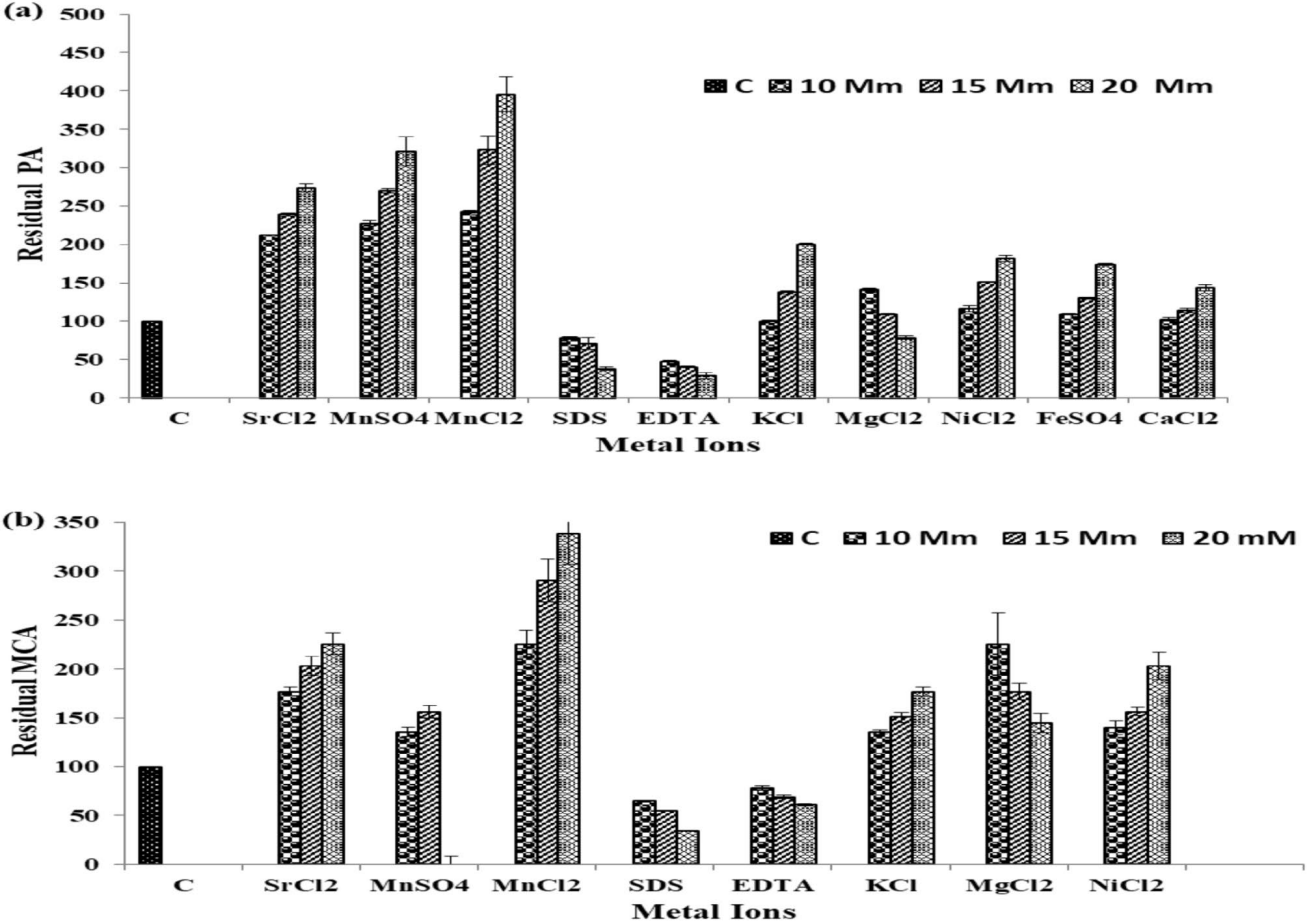
Effect of metal ions and inhibitors on purified enzyme activity: (a) PA and (b) MC activity in the presence of different compounds at the indicated concentrations.

This experiment was performed since many proteases, including those produced by *Bacillus* species, are metalloproteins, and activity is often dependent on the presence of specific metal ions (Mn^2+^, Ca^2+^, and Sr^2+^) (Saeed et al. 2023; Selvaraj et al. 2024). The rationale for testing these metal ions was to identify essential cofactors that improve enzyme performance. The effect of metal ions on the enzyme activity was assessed by testing various metal salts at concentrations ranging from 1 mM to 20 mM. Figure 6 shows the residual activity of the enzyme in the presence of different metal ions (MnCl_2_, MnSO_4_, CaCl_2_, ZnSO_4_, and HgSO_4_) at multiple concentrations. The results indicate that Mn^2+^ ions (both MnCl_2_ and MnSO_4_) significantly enhanced enzyme activity, with 2.2 times the control at 10 mM MnCl_2_ and four times at 20 mM MnSO_4_. In contrast, the presence of Hg^2+^ and Zn^2+^ resulted in a reduction of enzyme activity, with residual activities at 10 mM HgSO_4_ and ZnSO_4_, respectively. These findings demonstrate that Mn^2+^ plays a crucial role in enzyme activation, while Hg^2+^ and Zn^2+^ are strong inhibitors. The data were presented as mean ± SD shown in Figure 6.”

### 3.11 SDS–PAGE Analysis

SDS‐PAGE analysis was performed to assess the purity and molecular homogeneity of the enzyme (Figure S3). A prominent band corresponding to the expected molecular weight of alkaline proteases was observed, confirming successful purification and indicating that the enzyme comprises a single protein species (Hashmi et al. 2022). The presence of a single band suggests that the enzyme was purified to near‐homogeneity, with no significant contamination from other proteins (Tarek et al. 2023). The molecular weight of the band is consistent with previously reported values for alkaline proteases produced by *Bacillus* species (Ullah et al. 2022), further supporting the identity and purity of the enzyme preparation.

## 4 Discussion

The current study has magnificently proved the *B. stercoris* NCCP‐3139 as a strong source of alkaline protease with an effective MCA and thus exhibited its capability at industrial dairy applications. The taxonomic identity of the isolate was verified/confirmed using morphological, biochemical, and molecular analysis, while enzymatic activity identified its unique proteolytic and rennin‐like capabilities.

The Results of morphological and Gram‐staining showed distinctive *Bacillus* characteristics, irregular and opaque colonies and Gram‐positive and endospore‐forming rods, which were also reported by Acharjee et al. (2025) & Peng et al. (2025) in their previous study of *Bacillus* spp. in which the characteristics of the colonies of *B. subtilis* and *B. licheniformis* were similar, including their morphological appearances, and motility (Acharjee et al. 2025; Peng et al. 2025). The classification was also confirmed by the biochemical profiling as the isolate displayed positive MR, urease, catalase, and starch hydrolysis tests, which matched with the metabolic profile of *Bacillus* species, described by (Uddin et al. 2014). All these pooled characteristics robust *B. stercoris* as a strong proteolytic bacterium which can produce extracellular enzymes.

The high level of casein hydrolysis and significant milk‐clotting effect suggest the synthesis of extracellular rennin‐like protease. The Zhang, Tao, et al. (2025) and Zhang, Yang, et al. (2025) found similar results and claimed that *Bacillus cereus* and *B. subtilis* strains had a dual proteolytic/milk‐clotting protease that could be used in manufacturing cheese. The purification yielded a high MCA‐to‐PA ratio (550), which further indicates selective cleavage of κ‐casein, which is critical to the destabilization of microtubules by microbial rennin analogs (Jasim et al. 2025).

The analysis of the comparative milk composition showed significance (*p* < 0.05) interspecies differentiation, which has an effect on enzymatic performance. The sheep and buffalo milks had better proteolytic and clotting activities because of higher protein and casein levels, which are align with the results of Cecchinato et al. (2012) who highlighted that greater amounts of total solids and calcium content upwardly influence the efficiency of enzyme‐mediated coagulation (Cecchinato et al. 2012). In comparison with camel milk, camel milk was less active, which aligns with the lower casein micelle density and higher fraction of 2‐casein, which has been reported by (Ellouze et al. 2021). Ammonium sulfate precipitation, ion exchange, and gel filtration purification increased specific activity significantly (1.6 to 5.0 U/mg) as observed by Johnvesly and Naik (2001) in the *Bacillus flexus* protease purification (Johnvesly and Naik 2001). The increase in specific activity and the decrease in yield are the indicators of successful enrichment of catalytically active enzyme fractions. SDS‐PAGE showed one band of protein that matched the size of alkaline proteases (2535 kDa), which is expected considering the molecular weights of proteases secreted by *Bacillus* reported by (Winarti et al. 2018).

The finding of the study significantly demonstrated that, the alkaline protease as thermostable with optimal proteolytic and milk‐clotting activities at 55°C and pH 9.0. Similar results were observed by Pulikkottil Rajan (2024) and Ullah et al. (2022a), who found that the *Bacillus* strains alkaline proteins are most stable under moderate temperatures and alkalinity, and useful during their application in industrial practices such as dairy and detergent formulations (Pulikkottil Rajan 2024; Ullah et al. 2022). The rapid loss of activity at temperatures above 55°C and pH values above 9.0 is indicative of thermal denaturation or ionization of active‐site residues, a characteristic of serine proteases. Assays of metal ions demonstrated that Mn^2+^, Ca^2+^, and Sr^2+^ increased the enzyme activity, whereas EDTA and Hg^2+^ reduced it substantially, indicating the metalloprotein character of the enzyme. This result is consistent with that of Vazquez‐Armenta et al. (2022) and Krstić et al. (2005), who observed that Mn^2+^ plays a crucial role in stabilizing the enzyme's catalytic conformation (Vazquez‐Armenta et al. 2022; Krstić et al. 2005).

The observed activation by Mn^2+^ and inhibition by EDTA, together with the effects of other metal ions, suggests that the enzyme may be metal‐dependent. Similar response patterns have been reported for bacterial proteases, including *Bacillus*‐derived enzymes, in which Mn^2+^ enhanced activity while EDTA markedly reduced it (Lee et al. 2016). However, these observations are still indirect, and additional mechanistic evidence is required before the enzyme can be classified confidently as a metalloprotease. In particular, restoration of activity after EDTA treatment by re‐addition of specific metal ions, as well as direct metal‐binding assays, would provide stronger evidence for the role of Mn^2+^ or other cofactors in catalysis (Yu and Lee 1999; Yao et al. 2023). These approaches would help define the specific contribution of metal ions to enzyme activation and provide a more rigorous understanding of its catalytic mechanism.

This study adds to the growing body of research on microbial milk‐clotting enzymes by identifying *Bacillus stercoris* NCCP‐3139 as a source of a protease with relatively favorable clotting selectivity. In microbial coagulants, a higher MCA/PA ratio is generally considered desirable because it reflects stronger clotting performance with lower nonspecific proteolysis, an important feature for cheese manufacture (Narwal et al. 2017), (Zhang et al. 2019). Compared with previous reports on milk‐clotting proteases from *Bacillus subtilis* , *B. licheniformis* , *B. megaterium* , and *B. velezensis* , the present work contributes species‐specific evidence for *B. stercoris* and provides purification‐ and characterization‐based data that support its further evaluation as a microbial rennet candidate (Narwal et al. 2017; Yao et al. 2023). In addition, studies evaluating *Bacillus*‐derived milk‐clotting enzymes in cheese systems suggest that such enzymes can show practical promise, although performance depends strongly on enzyme specificity, casein hydrolysis pattern, and cheese type (Wehaidy et al. 2023; Mamo et al. 2020). Nevertheless, additional validation through kinetic analysis, direct κ‐casein cleavage studies, and cheese‐making trials will be necessary before industrial applicability can be established with confidence.

## 5 Conclusion

In this study, a new strain, *B. stercoris* NCCP‐3139, was identified and characterized; it produces a thermostable, alkaline, and metal‐dependent protease with considerable milk‐clotting capacity. Morphological, biochemical, and molecular studies revealed that it was taxonomically identical to *B. stercoris* JCM 30051 (98.75% similarity). The enzyme was strongly casein‐hydrolyzing and rennin‐like, with efficient cleavage of κ‐casein, an important step in micelle destabilization during cheese production. The process of sequential purification by ammonium sulfate precipitation, ion exchange, and gel filtration increased specific activity (5.0 U/mg) and MCA/PA ratio (550), proving the enrichment and selective behavior of the enzyme. Its thermostability and preference for alkaline was demonstrated by optimal activity at 55°C and pH 9.0. The addition of Mn^2+^ improved catalytic activity, whereas Hg^2+^, Zn^2+^, EDTA, and SDS suppressed it, thereby demonstrating its metalloprotein character. SDS‐PAGE analysis showed a single band, indicating the presence of the purified product. Further studies should be conducted on gene cloning, immobilization, and scale‐up bioprocessing to maximize enzyme stability, production, and industrial use in the dairy and biopharmaceutical industries. Future research should focus on pilot‐scale fermentation, gene cloning, enzyme immobilization, and cheese production trials to evaluate the industrial potential of the process.

## Author Contributions

**Muhammad Sibtain:** methodology, visualization, formal analysis, software, writing – review and editing, writing – original draft. **Ghulam Murtaza:** investigation, conceptualization, funding acquisition, visualization, writing – review and editing. **Sadaf Javaria:** conceptualization, investigation, visualization, validation, supervision, software. **Noor‐ul Ain:** investigation, validation, visualization. **Ashraf Ahmed Qurtam:** investigation, funding acquisition, validation, formal analysis, software, data curation. **Sadia Chaman:** investigation, validation, formal analysis, data curation. **Ali Zaman:** conceptualization, investigation, funding acquisition, writing – original draft, methodology, formal analysis, project administration, supervision, resources. **Mohammed Al‐zharani:** investigation, validation, software, data curation, resources, formal analysis. **Fahd A. Nasr:** investigation, validation, formal analysis, supervision.

## Funding

This work was supported and funded by the Deanship of Scientific Research at Imam Mohammad Ibn Saud Islamic University (IMSIU) (Grant number IMSIU‐DDRSP2601).

## Disclosure

The authors declare that no Generative AI was used in the creation of this manuscript.

## Conflicts of Interest

The authors declare no conflicts of interest.

## Supporting information


**Figure S1:** Morphological features of *B. stercoris* NCCP‐3139: (a) colony morphology, (b) Gram staining, (c) motility in semi‐solid agar.
**Figure S2:** Molecular characterization: (a) PCR amplification of 16S rRNA gene (~1116 bp), and phylogenetic tree.
**Figure S3:** SDS–PAGE Analysis of Purified Enzyme. Showing an indicator of the molecular weight (Ladder) (Lane 1), the ultracentrifugation retentate (Lane 2) and the Sephacryl S‐200‐purified enzyme (Lane 3).

## Data Availability

The original contributions presented in the study are included in the article/Supporting Information; further inquiries can be directed to the corresponding author.

## References

[fsn371864-bib-0001] Acharjee, S. A. , P. Bharali , D. Ramachandran , et al. 2025. “Bacterial Cell Factories for Sustainable Production of Green Polyesters Using Saccharides: Unlocking Potential to Mitigate Microplastic Pollution.” Journal of Polymer Research 32, no. 7: 1–22.

[fsn371864-bib-0002] Ali, T. H. , L. A. Mohamed , E. M. Abdellah , S. Farouk , and D. H. El‐Ghonemy . 2025. “Biochemical and Functional Characterization of a *Penicillium purpurescens* Milk‐Clotting Enzyme as an Animal Rennet Alternative.” World Academy of Sciences Journal 7, no. 6: 111.

[fsn371864-bib-0003] Al‐Joda, B. M. S. , and A. H. Jasim . 2021. “Biochemical Testing Revision for Identification of Several Kinds of Bacteria.” Journal of University of Babylon for Pure and Applied Sciences 29, no. 2: 168–176.

[fsn371864-bib-0004] Baggiolini, M. , J. Schnyder , U. Bretz , and B. Dewald . 2009. “Degradation of Extracellular Proteins.” Protein Degradation in Health and Disease 75: 105–121. 10.1002/9780470720585.ch7 399884

[fsn371864-bib-0005] Banerjee, G. , and A. K. Ray . 2017. “Impact of Microbial Proteases on Biotechnological Industries.” Biotechnology & Genetic Engineering Reviews 33, no. 2: 119–143. 29205093 10.1080/02648725.2017.1408256

[fsn371864-bib-0006] Ben Amira, A. , S. Besbes , H. Attia , and C. Blecker . 2017. “Milk‐Clotting Properties of Plant Rennets and Their Enzymatic, Rheological, and Sensory Role in Cheese Making: A Review.” International Journal of Food Properties 20, no. sup1: S76–S93.

[fsn371864-bib-0007] Bilal, M. , and H. M. N. Iqbal . 2020. “State‐of‐the‐Art Strategies and Applied Perspectives of Enzyme Biocatalysis in Food Sector Current Status and Future Trends.” Critical Reviews in Food Science and Nutrition 60, no. 12: 2052–2066. 31210055 10.1080/10408398.2019.1627284

[fsn371864-bib-0008] Bytyçi, P. , R. Hasalliu , M. Mestani , et al. 2025. “Physicochemical Properties of Milk and Aspects of Traditional Cheese in Kosovo.” Food Science and Technology 13, no. 1: 36–43.

[fsn371864-bib-0009] Cecchinato, A. , M. Penasa , C. C. Gotet , M. De Marchi , and G. Bittante . 2012. “Factors Affecting Coagulation Properties of Mediterranean Buffalo Milk.” Journal of Dairy Science 95, no. 4: 1709–1713. 22459819 10.3168/jds.2011-4694

[fsn371864-bib-0010] Chen, C. C. , L. Y. Chen , W. T. Li , et al. 2021. “Influence of Chymosin on Physicochemical and Hydrolysis Characteristics of Casein Micelles and Individual Caseins.” Nanomaterials 11, no. 10: 2594. 34685035 10.3390/nano11102594 PMC8539682

[fsn371864-bib-0011] Chouaia, B. , and J. Dittmer . 2024. “A 2000‐Year‐Old *Bacillus stercoris* Strain Sheds Light on the Evolution of Cyclic Antimicrobial Lipopeptide Synthesis.” Microorganisms 12, no. 2: 338. 38399742 10.3390/microorganisms12020338 PMC10893106

[fsn371864-bib-0012] Ellouze, M. , C. Vial , H. Attia , and M. A. Ayadi . 2021. “Effect of pH and Heat Treatment on Structure, Surface Characteristics and Emulsifying Properties of Purified Camel β‐Casein.” Food Chemistry 365: 130421. 34216912 10.1016/j.foodchem.2021.130421

[fsn371864-bib-0013] Hashmi, S. , S. Iqbal , I. Ahmed , and H. A. Janjua . 2022. “Production, Optimization, and Partial Purification of Alkali‐Thermotolerant Proteases From Newly Isolated *Bacillus subtilis* S1 and *Bacillus amyloliquefaciens* KSM12.” Pro 10, no. 6: 1050.

[fsn371864-bib-0014] Ibrahim, E. N. , and K. Ma . 2017. “Industrial Applications of Thermostable Enzymes From Extremophilic Microorganisms.” Current Biochemical Engineering 4, no. 2: 75–98.

[fsn371864-bib-0015] Im, A. E. , D. Bharti , G. Lim , et al. 2025. “Low‐Grade Green Coffee With *Bacillus amyloliquefaciens* NY124 Protease: Extraction, Purification, and Functional Synergy of Chlorogenic Acid and Trigonelline.” AMB Express 15, no. 1: 138. 41023539 10.1186/s13568-025-01920-7 PMC12480324

[fsn371864-bib-0016] Jasim, B. H. , K. R. Mohammed , S. A. Mohammed , S. D. Sulaiman , and A. F. Hasan . 2025. “Extraction, Optimization, and Purification of Anti‐Cancer L‐Glutaminase Enzyme From *Bacillus subtilis* B7.” Opera Medica et Physiologica 12, no. 2: 43–57.

[fsn371864-bib-0017] Johnvesly, B. , and G. R. Naik . 2001. “Studies on Production of Thermostable Alkaline Protease From Thermophilic and Alkaliphilic *Bacillus* sp. JB‐99 in a Chemically Defined Medium.” Process Biochemistry 37, no. 2: 139–144.

[fsn371864-bib-0018] Khatoon, N. , N. Ullah , A. Sarwar , et al. 2023. “Isolation and Identification of Protease‐Producing *Bacillus* Strain From Cold Climate Soil and Optimization of Its Production by Applying Different Fermentation Conditions.” Applied Ecology and Environmental Research 21, no. 4: 3391–3401.

[fsn371864-bib-0019] Krstić, D. , K. Krinulović , and V. Vasić . 2005. “Inhibition of Na^+^/K^+^‐ATPase and Mg^2+^‐ATPase by Metal Ions and Prevention and Recovery of Inhibited Activities by Chelators.” Journal of Enzyme Inhibition and Medicinal Chemistry 20, no. 5: 469–476. 16335055 10.1080/14756360500213280

[fsn371864-bib-0020] Kumar, H. , S. Guleria , K. Kuča , et al. 2025. “Emerging Prospects of *Bacillus* Species in Attaining the Sustainable Development Goals.” Applied Microbiology and Biotechnology 109, no. 1: 242. 41175226 10.1007/s00253-025-13634-8 PMC12579670

[fsn371864-bib-0021] Lee, E. , H. Lee , D. Lee , and H. Kim . 2016. “Characterization of a Metalloprotease From an Isolate *Bacillus thuringiensis* 29‐126 in Animal Feces Collected From a Zoological Garden in Japan.” Journal of Applied Biological Chemistry 59: 373–377. 10.3839/jabc.2016.063.

[fsn371864-bib-0022] Mamo, J. , P. Getachew , M. Kuria , and F. Assefa . 2020. “Application of Milk‐Clotting Protease From Aspergillus Oryzae DRDFS13 MN726447 and *Bacillus subtilis* SMDFS 2B MN715837 for Danbo Cheese Production.” Journal of Food Quality 2020: 1–12. 10.1155/2020/8869010.

[fsn371864-bib-0023] Moschopoulou, E. 2017. “Microbial Milk Coagulants.” In Microbial Enzyme Technology in Food Applications, 199–213. CRC Press.

[fsn371864-bib-0024] Nair, A. V. , N. K. Praveen , N. Joseph , A. M. Leo , and K. K. Vijayan . 2021. “Isolation and Characterization of a Novel Antimicrobial Oxatetracyclo Ketone From *Bacillus stercoris* MBTDCMFRI Ba37 Isolated From the Tropical Estuarine Habitats of Cochin.” Molecular Biology Reports 48, no. 2: 1299–1310. 33590414 10.1007/s11033-021-06146-x

[fsn371864-bib-0025] Narwal, R. , B. Bhushan , A. Pal , and S. Malhotra . 2017. “Optimization of Upstream Process Parameters for Enhanced Production of Thermostable Milk Clotting Enzyme From *Bacillus subtilis* MTCC 10422.” Journal of Food Process Engineering 40: 12356. 10.1111/jfpe.12356.

[fsn371864-bib-0026] Naveed, M. , Y. Wang , X. Yin , et al. 2023. “Purification, Characterization and Bactericidal Action of Lysozyme, Isolated From *Bacillus subtillis* BSN314: A Disintegrating Effect of Lysozyme on Gram‐Positive and Gram‐Negative Bacteria.” Molecules 28, no. 3: 1058. 36770725 10.3390/molecules28031058 PMC9919333

[fsn371864-bib-0027] Patil, R. C. , and B. L. Jadhav . 2017. “Isolation and Characterization of Protease Producing *Bacillus* Species From Soil of Dairy Industry.” International Journal of Current Microbiology and Applied Sciences 6, no. 6: 853–860.

[fsn371864-bib-0028] Peng, W. , H. Xu , M. Zhang , B. Xu , B. Dai , and C. Yang . 2025. “Effects of *Bacillus licheniformis* on Growth, Biofilm, Motility, and Quorum Sensing of *Salmonella typhimurium* .” Microorganisms 13, no. 7: 1540. 40732049 10.3390/microorganisms13071540 PMC12299142

[fsn371864-bib-0029] Priyashantha, H. 2025. “World Dairy System Sustainability: A Milk Quality Perspective.” Frontiers in Sustainable Resource Management 4: 1572962.

[fsn371864-bib-0030] Pulikkottil Rajan, D. 2024. “Extraction, Isolation, and Characterization Techniques of Structural Proteins.” In Fish Structural Proteins and Its Derivatives: Functionality and Applications, 37–72. Springer.

[fsn371864-bib-0031] Raman, J. , J. S. Noh , J. S. Kim , et al. 2025. “Role of *Bacillus* Species in Food Industry: Advantages and Limitations.” Journal of Microbiology and Biotechnology 35: e2507043. 41162176 10.4014/jmb.2507.07043 PMC12603379

[fsn371864-bib-0032] Rath, S. , and S. Das . 2023. “Oxidative Stress‐Induced DNA Damage and DNA Repair Mechanisms in Mangrove Bacteria Exposed to Climatic and Heavy Metal Stressors.” Environmental Pollution 339: 122722. 37863253 10.1016/j.envpol.2023.122722

[fsn371864-bib-0033] Ryu, S. W. , J. C. Moon , B. S. Oh , et al. 2024. “Anti‐Obesity Activity of Human Gut Microbiota *Bacteroides stercoris* KGMB02265.” Archives of Microbiology 206, no. 1: 19. 10.1007/s00203-023-03750-2 38086977

[fsn371864-bib-0034] Sacchi, C. T. , A. M. Whitney , L. W. Mayer , et al. 2002. “Sequencing of 16S rRNA Gene: A Rapid Tool for Identification of *Bacillus anthracis* .” Emerging Infectious Diseases 8, no. 10: 1117–1123. 12396926 10.3201/eid0810.020391 PMC2730316

[fsn371864-bib-0035] Saeed, K. , S. Riaz , A. Adil , et al. 2023. “Characterization of Alkaline Metalloprotease Isolated From Halophilic Bacterium *Bacillus cereus* and Its Applications in Various Industrial Processes.” Anais da Academia Brasileira de Ciências 95: e20230014. 37878911 10.1590/0001-3765202320230014

[fsn371864-bib-0036] Satilmis, S. , N. U. Toprak , C. Ilgın , and G. Soyletir . 2019. “Evaluation of Direct 16S rRNA PCR From Clinical Samples for Bacterial Detection in Normally Sterile Body Sites.” Journal of Infection in Developing Countries 13, no. 11: 978–983. 32087069 10.3855/jidc.11732

[fsn371864-bib-0037] Selvaraj, C. , P. Vijayalakshmi , A. M. Alex , A. S. Alothaim , R. Vijayakumar , and V. R. Umapathy . 2024. “Metalloproteins Structural and Functional Insights Into Immunological Patterns.” Advances in Protein Chemistry and Structural Biology 141: 67–86. 38960487 10.1016/bs.apcsb.2024.03.009

[fsn371864-bib-0038] Stocco, G. , C. Cipolat‐Gotet , A. Summer , et al. 2025. “Modeling the Relationships Among Technological Properties of Sheep Milk, Animal Factors, Composition, and Minerals Using Generalized Additive Mixed Models.” Journal of Dairy Science 108, no. 4: 3334–3353. 40043759 10.3168/jds.2024-25846

[fsn371864-bib-0039] Suryani, E. M. , Y. D. Jatmiko , and I. Mustafa . 2023. “Detection of Plantaricin‐Encoding Gene and Its Partial Purification in *Lactobacillus plantarum* BP102.” Jurnal Biodjati 8, no. 2: 233–247.

[fsn371864-bib-0040] Taredahalli, N. 2013. Isolation, Characterization and Evaluation of Bacillus spp. Infective to White Grubs. Scarabaeidae.

[fsn371864-bib-0041] Tarek, H. , K. B. Nam , Y. K. Kim , S. A. Suchi , and J. C. Yoo . 2023. “Biochemical Characterization and Application of a Detergent Stable, Antimicrobial and Antibiofilm Potential Protease From *Bacillus siamensis* .” International Journal of Molecular Sciences 24, no. 6: 5774. 36982846 10.3390/ijms24065774 PMC10056560

[fsn371864-bib-0042] Uddin, M. E. , P. Maitra , H. M. Faruquee , and M. F. Alam . 2014. “Isolation and Characterization of Protease Enzyme From Locally Isolated *Bacillus* sp.” American Journal of Life Sciences 2, no. 6: 338–344.

[fsn371864-bib-0043] Ullah, N. , M. U. Rehman , A. Sarwar , et al. 2022. “Purification, Characterization, and Application of Alkaline Protease Enzyme From a Locally Isolated *Bacillus cereus* Strain.” Fermentation 8, no. 11: 628.

[fsn371864-bib-0044] Vazquez‐Armenta, F. J. , U. F. Valdez‐Olmos , A. A. Arvizu‐Flores , J. F. Ayala‐Zavala , A. Ochoa‐Leyva , and A. A. Lopez‐Zavala . 2022. “Metal Ions and Chemical Modification Reagents Inhibit the Enzymatic Activity of Lecithin‐Dependent Hemolysin From *Vibrio parahaemolyticus* .” Toxins 14, no. 9: 609. 36136547 10.3390/toxins14090609 PMC9506434

[fsn371864-bib-0045] Visentin, G. , G. Visentin , M. Marchi , et al. 2017. “Factors Associated With Milk Processing Characteristics Predicted by Mid‐Infrared Spectroscopy in a Large Database of Dairy Cows.” Journal of Dairy Science 100, no. 4: 3293–3304. 10.3168/jds.2016-12028. 28131580

[fsn371864-bib-0046] Wang, H. , J. Wei , Z. Yang , et al. 2025. “Purification and Identification of an Antimicrobial Protein From *Bacillus stercoris* TY‐12 and Its Biocontrol Functions Against *Ralstonia solanacearum* .” Applied Microbiology 5, no. 1: 2.

[fsn371864-bib-0047] Wehaidy, H. , M. Abdel‐Naby , A. Kholif , M. Elaaser , W. Bahgaat , and W. Wahab . 2023. “The Catalytic and Kinetic Characterization of *Bacillus subtilis* MK775302 Milk Clotting Enzyme: Comparison With Calf Rennet as a Coagulant in White Soft Cheese Manufacture.” Journal, Genetic Engineering & Biotechnology 21: 61. 10.1186/s43141-023-00513-w. PMC10192502 37195386

[fsn371864-bib-0048] Winarti, A. , N. A. Fitriyanto , A. Pertiwiningrum , Z. Bachruddin , Y. Pranoto , and Y. Erwanto . 2018. “Optimization of Protease Purification From *Bacillus cereus* TD5B by Ammonium Sulfate Precipitation.” Chemical Engineering Transactions 63: 709–714.

[fsn371864-bib-0049] Yao, Z. , J. Hua , J. Wanga , et al. 2023. “Purification and Characteristics of a Novel Milk‐Clotting Metalloprotease From *Bacillus velezensis* DB219.” Journal of Dairy Science 106: 6688–6700. 10.3168/jds.2023-23450. 37558047

[fsn371864-bib-0050] Yu, M. , and C. Lee . 1999. “Expression and Characterization of the prtV Gene Encoding a Collagenase From *Vibrio parahaemolyticus* in *Escherichia coli* .” Microbiology 145, no. Pt 1: 143–150. 10.1099/13500872-145-1-143. 10206692

[fsn371864-bib-0051] Zhang, T. , Z. Yang , Y. Zhang , et al. 2025. “Proteomics‐Guided Isolation of a Novel Serine Protease With Milk‐Clotting Activity From Tamarillo ( *Solanum betaceum* Cav.).” Food Chemistry 465: 141956. 39541676 10.1016/j.foodchem.2024.141956

[fsn371864-bib-0052] Zhang, X. , L. Tao , G. Wei , et al. 2025. “Plant‐Derived Rennet: Research Progress, Novel Strategies for Their Isolation, Identification, Mechanism, Bioactive Peptide Generation, and Application in Cheese Manufacturing.” Critical Reviews in Food Science and Nutrition 65, no. 3: 444–456. 37902764 10.1080/10408398.2023.2275295

[fsn371864-bib-0053] Zhang, Y. , Y. Xia , P. Lai , et al. 2019. “Fermentation Conditions of Serine/Alkaline Milk‐Clotting Enzyme Production by Newly Isolated *Bacillus licheniformis* BL312.” Annals of Microbiology 69: 1289–1300. 10.1007/s13213-019-01513-3.

