# Supplementary material for: Milk‐Clotting Protease From Bacillus stercoris NCCP‐3139: A Potential Microbial Rennet for Cheese Production
Source: Food Sci Nutr. 2026 May 13;14(5):e71864. doi: 10.1002/fsn3.71864 (PMC13172265; doi:10.1002/fsn3.71864)
Supplement: Supplementary file 1 — Figure S1: Morphological features of B. stercoris NCCP‐3139: (a) colony morphology, (b) Gram staining, (c) motility in semi‐solid agar. Figure S2: Molecular characterization: (a) PCR amplification of 16S rRNA gene (~1116 bp), and phylogenetic tree. Figure S3: SDS–PAGE Analysis of Purified Enzyme. Showing an indicator of the molecular weight (Ladder) (Lane 1), the ultracentrifugation retentate (Lane 2) and the Sephacryl S‐200‐purified enzyme (Lane 3). [file FSN3-14-e71864-s001.docx]

**Milk-Clotting Protease from Bacillus stercoris NCCP-3139: A Potential Microbial Rennet for Cheese Production**

Muhammad Sibtain^1^, Sadaf Javaria^1^, Ghulam Murtaza^2^, Sadia Chaman^3^, Noor-ul-ain^1^, Ali Zaman^3*^Adnan Amin^4*^

^1^ Institute of Food Science and Nutrition, Gomal University, Dera Ismail Khan.

^2^Faculty of Veterinary and Animal Sciences, Gomal University, Dera Ismail Khan.

^3^ Institute of Pharmaceutical Sciences, UVAS, Lahore, Pakistan.

^4^ Institute of Microbiology, Gomal University, Dera Ismail Khan.

^5^ Department of Life Sciences, Yeungnam University, Gyeongsan 38541, Republic of South Korea,

Corresponding Author:

alizaman@gu.edu.pk, adnan.amin@yu.ac.kr


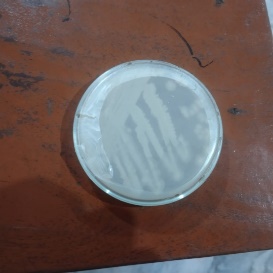

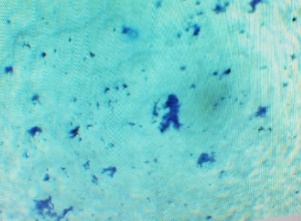


**(a)**

**(b)**


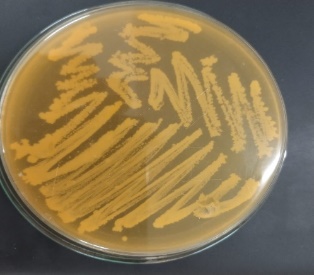


**(c)**

**Fig. S1:** Morphological features of *B. stercoris* NCCP-3139: (a) colony morphology, (b) Gram staining, (c) motility in semi-solid agar.


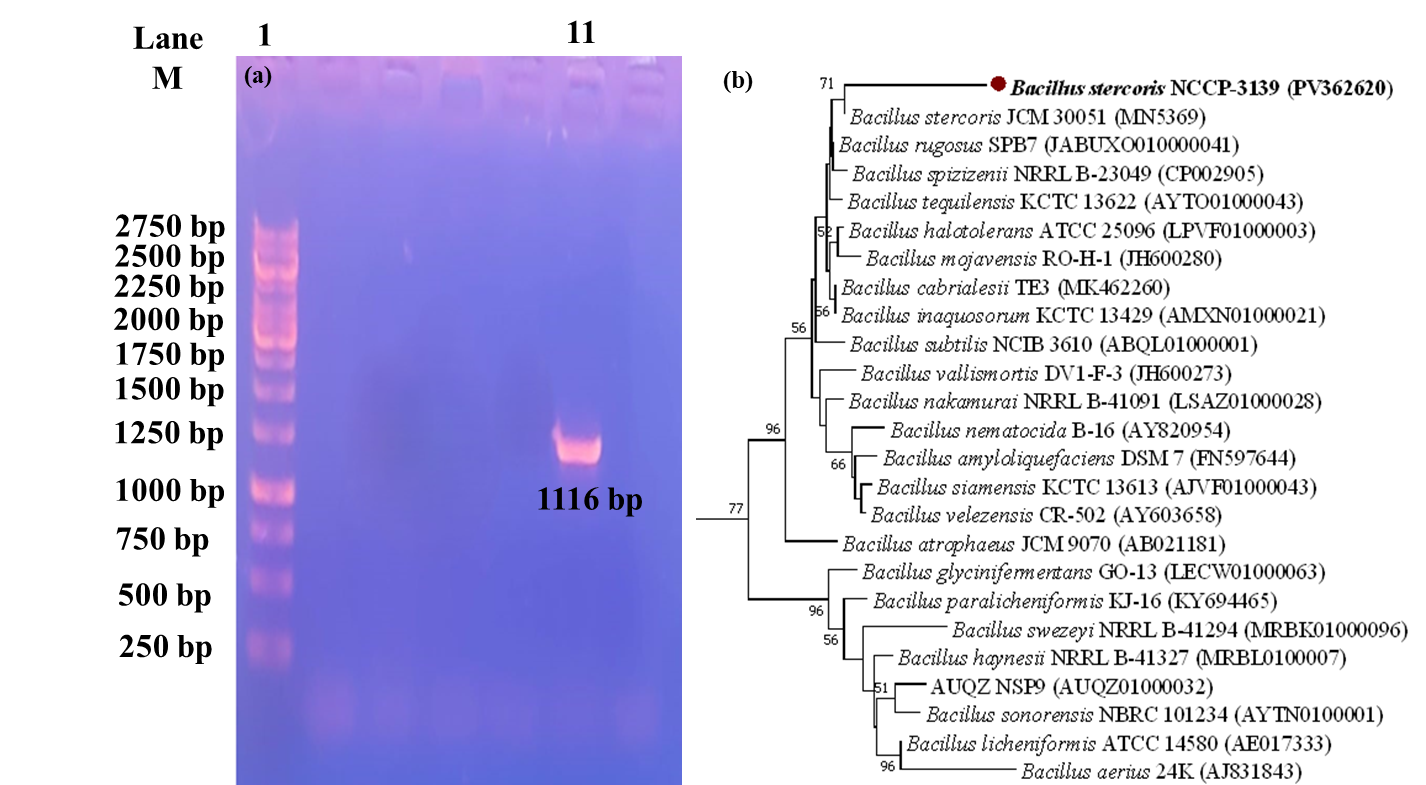


**Figure S2:** : Molecular characterization: (a) PCR amplification of 16S rRNA gene (~1116 bp), and phylogenetic tree.

**
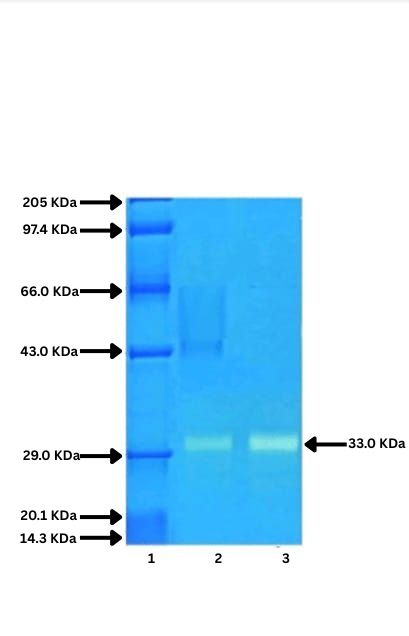
**

**Figure S3:** SDS–PAGE Analysis of Purified Enzyme. Showing an indicator of the molecular weight (Ladder)(Lane 1), the ultracentrifugation retentate (Lane 2) and the Sephacryl S-200-purified enzyme (Lane 3 ).
